# The Importance of Substituent Position for Antibacterial Activity in the Group of Thiosemicarbazide Derivatives

**DOI:** 10.3390/molecules29061333

**Published:** 2024-03-17

**Authors:** Sara Janowska, Joanna Stefańska, Dmytro Khylyuk, Monika Wujec

**Affiliations:** 1 Department of Pathobiochemistry and Interdisciplinary Applications of Ion Chromatography, Biomedical Sciences, Medical University of Lublin, 1 Chodzki Street, 20-093 Lublin, Poland; 2 Department of Pharmaceutical Microbiology, Centre for Preclinical Research, Medical University of Warsaw, Banacha 1B Street, 02-097 Warsaw, Poland; joanna.stefanska@wum.edu.pl; 3 Department of Organic Chemistry, Faculty of Pharmacy, Medical University, 4a Chodzki Street, 20-093 Lublin, Poland; dmytro.khylyuk@umlub.pl

**Keywords:** synthesis of thiosemicarbazide, structure-activity relationship, antibacterial activity

## Abstract

The search for new antibacterial compounds is still a huge challenge for scientists. Each new chemotherapy drug is not 100% effective when introduced into treatment. Bacteria quickly become resistant to known structures. One promising group of new compounds is thiosemicarbazides. In the presented work, we looked for the relationship between structure and antibacterial activity within the group of thiosemicarbazide derivatives. This is a continuation of our previous work. Here, we decided to check to what extent the position of the 3-methoxyphenyl substituent affects potency. We obtained new structures that differ in the positions of the substituent in the thiosemicarbazide skeleton. Based on the obtained results of the biological tests, it can be concluded that the substituent in position 1 of thiosemicarbazide derivatives significantly determines their activity. Generally, among the substituents used, trifluoromethylphenyl turned out to be the most promising. The MIC values for compounds with this substituent are 64 µg/mL towards *Staphylococci* sp. Using molecular docking, we tried to explain the mechanism behind the antibacterial activity of the tested compounds.

## 1. Introduction

Throughout history, infectious diseases caused by bacteria have been a threat to public health, accounting for a significant number of illnesses and high mortality rates worldwide. In 1942, there was a breakthrough in the fight against pathogenic microorganisms—the first antibiotic, penicillin, was introduced into clinical practice. Since then, doctors and scientists have witnessed the phenomenon of microorganisms quickly becoming resistant to the effects of bactericidal agents [1]. Factors stimulating this drastic increase in pathogen resistance to known medicinal substances are the large number of antibiotics used, patients’ easy access to these drugs, and their overuse [2,3]. Hospitals are a particularly risky environment for the development of antibiotic resistance in pathogens, where drug-resistant strains of bacteria gradually become resistant to all known antibacterial drugs [4,5]. The response to this phenomenon is the constant design and research of new drugs that differ from previous ones in terms of their chemical structure and mechanism of action. Unfortunately, nowadays, despite the discovery of many new antibiotics and chemotherapeutic agents, bacterial resistance to substances from standard classes of antibacterial drugs is constantly increasing [4]. This justifies the need to continue searching for pharmacological agents that would be effective against drug-resistant strains of bacteria common in hospital environments.

One group of substances among which it is justified to search for new compounds with possible uses in the treatment of infectious diseases is thiosemicarbazide derivatives. There are many research studies showing their anti-tuberculosis [6], antiviral [7], and antibacterial properties [8,9,10,11,12,13]. Compounds from the group of thiosemicarbazide derivatives are synthesized not only as potential bioactive molecules but also as precursors for the synthesis of heterocyclic compounds, such as thiadiazoles, oxadiazoles, and triazoles. Chemically, thiosemicarbazides are the simplest hydrazine derivatives of thiocarbamic acid. They contain the NH_2_-NH-C(=S)NH_2_ group in their structure, thus constituting them sulfur equivalents of semicarbazide functionality [14]. This structure provides thiosemicarbazide derivatives versatile applications in the synthesis of heterocyclic compounds and biological properties different from those of semicarbazide [15]. Molecules based on the thiosemicarbazide moiety are characterized by a different chemical structure from known antibacterial drugs. For this reason, the group of compounds we selected as our research material has the potential to be used as a new type of antimicrobial agents effective in the fight against infectious diseases caused by drug-resistant bacteria [13].

In recent years, our team has conducted several studies on the antibacterial potential of thiosemicarbazides [11]. These works show that these compounds have a certain therapeutic potential related to their mechanism of action, which is based on the inhibition of type IIA topoisomerase (DNA gyrase and topoisomerase IV) [11]. In this way, the actions of these compounds disrupt the synthesis of bacterial DNA, leading to inhibition of their replication. Continuing our previous work on the relationship between antibacterial activity and chemical structure in the thiosemicarbazide group, we decided to synthesize and test the antimicrobial activity of a series of new arylthiosemicarbazide derivatives.

**Figure 1:**
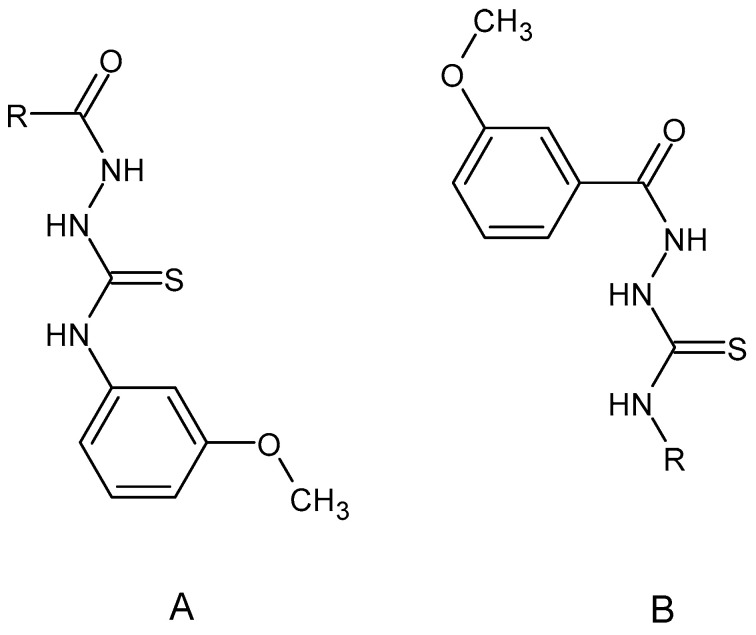
The general structure of thiosemicarbazide. Derivatives with structure (**B**) were obtained in previous work. Derivatives with structure (**A**) are the subject of this work.

In our recent work, we showed that thiosemicarbazides with a 3-methoxyphenyl substituent in position 1 and a phenyl ring with electron-donating and withdrawing substituents in position 4 (Figure 1B) have higher antibacterial activity than their cyclic analogs, such as 1,3,4-thiadiazoles [11]. At the same time, we observed a beneficial effect of the presence of halophenyl substituents on the molecule’s biological potential [11]. In this work, we decided to focus on the importance of the position of the 3-methoxyphenyl group within the thiosemicarbazide system for the antimicrobial activity of the molecule. For this purpose, we designed a series of structures with a 3-methoxyphenyl substituent in the 4-position of the thiosemicarbazide and a phenyl ring with a halogen substituent in the 1-position (Figure 1A). This made it possible to compare two series of compounds and consequently search for a relationship between structure and activity in several tested compounds.

A series of thiosemicarbazides were synthesized from the appropriate hydrazide and isothiocyanate using a conventional method. The structure of the obtained molecules was confirmed by nuclear magnetic resonance (NMR) spectra as well as elemental analyses. The antibacterial activity of the newly synthesized compounds was determined in vitro using the broth microdilution method against reference strains of Gram-negative and Gram-positive bacteria.

## 2. Results and Discussion

### 2.1. Chemistry

**Scheme 1:**
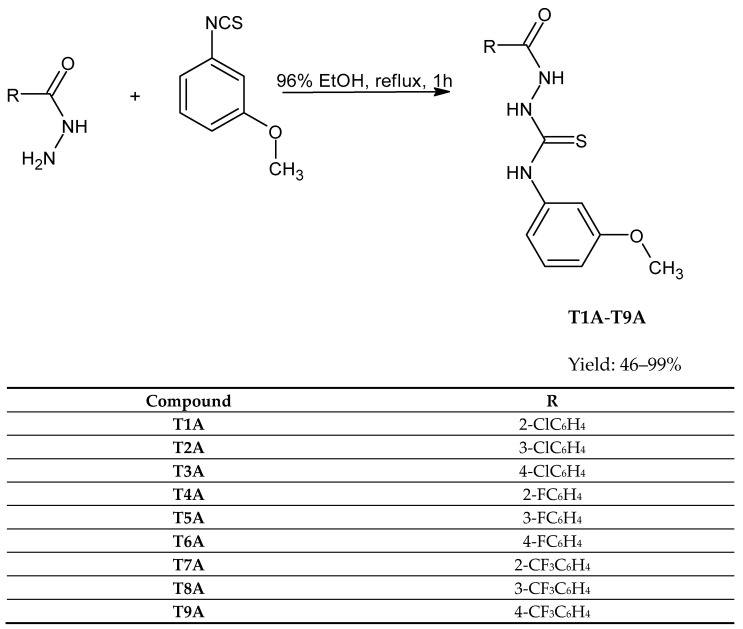
The synthesis pathway of thiosemicarbazides **T1A**–**T9A**.

The synthesis of thiosemicarbazides was carried out according to the synthetic path presented in Scheme 1. The tested compounds include both newly synthesized molecules (**T4A**, **T5A**, **T8A**) and previously obtained derivatives (**T1A**, **T2A**, **T3A**, **T6A**, **T7A**, **T9A**) [16,17].

The reaction between the appropriate hydrazides and 3-methoxyphenyl isothiocyanate in boiling ethanol afforded 1-aryl-4-(3-methoxyphenyl) thiosemicarbazides (**T1A**–**T9A**) in good yields (46–99%).

The structures of the new compounds, described for the first time, were determined using ^1^H NMR and ^13^C NMR spectroscopy and elemental analyses.

In the IR spectra of the newly synthesized compounds, absorption bands for C=O and C=S, confirming the formation of a thiosemicarbazide moiety in the molecule, were observed at 1638–1670 cm^−1^ and at 1361–1365 cm^−1^, respectively. The ^1^H NMR spectra showed chemical shifts of protons located on nitrogen atoms as two singlets, one in the range of 9.77–9.84 ppm corresponding to two protons and the second in the range of 10.33–10.82 ppm for one proton. Signals of three protons of the methoxy group at 3.75 ppm appearing as a singlet were observed. In the ^13^C NMR spectra, signals characteristic of individual carbon atoms and signals as doublets or quartets characteristic for carbon atoms bonded to the fluorine atom were observed.

IR, ^1^H NMR, and ^13^C NMR spectra for the new compounds are presented in the Supplementary Materials.

### 2.2. Antibacterial Evaluation

The antibacterial activity of the obtained thiosemicarbazides was tested by determining their ability to inhibit bacterial growth in vitro using the broth microdilution method. The results of the antibacterial potential tests (MIC value) are presented in Table 1.

The research was conducted on Gram-positive (*Staphylococcus aureus* NCTC 4163, *Staphylococcus* aureus ATCC 25923, *Staphylococcus* aureus ATCC 6538, *Staphylococcus* aureus ATCC 29213, *Staphylococcus epidermidis* ATCC 12228, *Staphylococcus epidermidis* ATCC 35984) and Gram-negative (*Escherichia coli* ATCC 25922, *Pseudomonas aeruginosa* ATCC 15442) bacterial strains. 

All synthesized thiosemicarbazide derivatives showed no antimicrobial activity against reference strains of Gram-negative, oxidase–negative bacteria *Escherichia coli* ATCC 25922, and oxidase–positive bacteria *Pseudomonas aeruginosa* ATCC 15442.

According to the results of our screening tests, some of the tested thiosemicarbazides showed potential antimicrobial activity against selected Gram-positive bacteria, including Gram-positive strains of *S. aureus* NCTC 4163, *S. aureus* ATCC 25923, *S. aureus* ATCC 6538, *S. aureus* ATCC 29213, and *S. epidermidis* ATCC 12228. The bacteriostatic activity of the synthesized thiosemicarbazides against these bacterial strains was in the range of 32–256 µg/mL. The drug used in our study was ciprofloxacin. The MIC values against Gram-positive bacteria for this antibiotic ranged from 0.125 to 0.5 µg/mL.

Within the studied group, the compounds differed in the type and position of the substituent on the phenyl ring located in position 1 towards the thiosemicarbazide moiety. This made it possible to trace the correlation between the antibacterial potential shown in the tests and the structural differences in this group of compounds.

According to our results, thiosemicarbazide **T4A**, having a fluorine substituent in the *ortho* position in the phenyl ring, showed the highest antibacterial activity against four bacterial strains of *Staphylococci* (*S. aureus* NCTC 4163, *S. aureus* ATCC 25923, *S. aureus* ATCC 6538, *S. aureus* ATCC 29213) with MIC values in the range of 32–64 µg/mL. At the same time, the remaining fluorophenyl derivatives (*meta* and *para*) showed very low bacteriostatic activity against all microorganisms used in the tests, including *S. aureus* strains.

All tested thiosemicarbazides containing a trifluoromethylphenyl moiety in their structure showed moderate bacteriostatic activity against four bacterial strains of *S. aureus*, with MIC values in the range of 64–128 µg/mL. This indicates the importance of the trifluoromethyl group as a pharmacophore determining antimicrobial activity in the group of thiosemicarbazide derivatives.

The lowest bacteriostatic activity against the *S. aureus* strains used in the tests was observed for compounds with a chlorophenyl moiety, with MIC values above 256 µg/mL, except for the *para* derivative, in which the values against four bacterial strains of *S. aureus* were in the range of 64–128 µg/mL.

Most of the tested compounds did not show bacteriostatic activity against the two strains of *S. epidermidis* used in the tests. The highest antibacterial potential among the tested molecules was demonstrated by thiosemicarbazide **T9A**, which has a trifluoromethyl group in the *para* position in the phenyl ring. The MIC values for this compound were 64 µg/mL against *S. epidermidis* ATCC 12228 and 128 µg/mL against *S. epidermidis* ATCC 35984.

All tested molecules had a 3-methoxyphenyl group located in position 4 of the thiosemicarbazides. The results obtained from the current series can be compared with data from a previous work published by our team, in which we examined the antibacterial activity of thiosemicarbazide derivatives having a 3-methoxyphenyl moiety in position 1 [11]. In both series, the molecules showed no antimicrobial activity against Gram-negative bacteria. At the same time, some of the compounds showed potential moderate activity against Gram-positive bacteria, particularly *S. aureus* and *S. epidermidis*. The comparison of the activity of the two series against comparable bacterial strains is presented in Table 2.

Comparing the data from the two series, it can be seen that most of the molecules having a 3-methoxyphenyl moiety in position 4 towards the thiosemicarbazide system show much higher antibacterial activity against the *S. aureus* ATCC 25923 strain than their analogs with a 3-methoxyphenyl moiety in position 1. For example, all derivatives with a trifluoromethylphenyl substituent from the new A series (**T7A**–**T9A**) showed activity against *S. aureus* with an MIC value of 64 µg/mL. In the case of their B series analogs, the MIC values against the same bacterial strain were in the range of 128–250 µg/mL.

In the case of the activity of thiosemicarbazide derivatives against *S. epidermidis* ATCC 12228, compounds from the B series with a trifluoromethylphenyl moiety in position 4 and a 3-methoxyphenyl moiety in position 1 (**T7B**–**T9B**) seem to have a more favorable bactericidal potential than the others. MIC values for these compounds ranged from 31.25 to 125 µg/mL. For comparison, the MIC values of all compounds from series A ranged from 128 to above 256 µg/mL.

In summary, the obtained results indicate that in the case of thiosemicarbazide derivatives, placing a 3-methoxyphenyl group in the 4-position and a phenyl ring with an attached halogen substituent in the 1-position is beneficial for selective bacteriostatic activity directed against *Staphylococcus aureus*.

When comparing the activity of compounds containing a chlorine atom in the phenyl ring, it can be concluded that only one derivative of the A series is more active than the same derivative of the B series. Among the compounds with a fluorine atom, all compounds of the A series turned out to be more active than the series B compounds. The greatest increase in activity was observed in the case of the *ortho* isomer, up to 5-fold. In the case of the *meta* and *para* isomers, it was 2-fold. The greatest increase in activity was observed in the case of compounds with a trifluoromethylphenyl substituent. Each of the A series compounds was four to eight times more active than the B series.

To sum up, only three new compounds were obtained in this study, but the tests were aimed to demonstrate differences in antibacterial activity between compounds differing in the position of substituents in relation to the thiosemicarbazide skeleton. Additionally, we wanted to demonstrate differences in the activity of compounds differing both in the type of substituent in the phenyl ring (chlorine and fluorine atoms, trifluoromethylphenyl group) in position 1 and its position in this ring. The antibacterial activity of the obtained derivatives varies widely. Among the isomers, in the case of compounds with fluorine, the *ortho* isomer was the most active, followed by the *meta* and least active *para*. When analyzing compounds containing a trifluoromethylphenyl group, the dependence of activity on the position of the substituent changes in a series *para* > *ortho* > *meta*. Among the derivatives with a chlorine atom, the most active is the *para* isomer, followed by the less active *meta* and *ortho* isomers.

### 2.3. Docking

Docking simulations demonstrated significant binding energies to Topoisomerase IV for both active and inactive compounds, comparable to those of the reference ligand (Table 3). This observation may be attributed to the inability to form stable complexes, due to the high flexibility inherent to thiosemicarbazides. In addition, docking simulations employing the same model revealed enhanced binding energies for **T3A** and **T4A** with DNA gyrase, slightly surpassing those of the reference ligand. Additionally, the docking scores indicated superior affinities of **T9A** towards Ddl in comparison to other tested compounds. These findings suggest a rather complex interaction effect exerted by the compounds, with certain enzymes displaying marked sensitivity.

Moreover, the results demonstrate that fluorine derivatives exhibit greater affinities than their chlorine counterparts. The presence of a fluorine substituent appears to augment binding energies, potentially due to an increased capacity for forming different types of interactions, like hydrogen bonds, halogen interactions, and stabilization of the ligand inside the binding site. 

**Figure 2:**
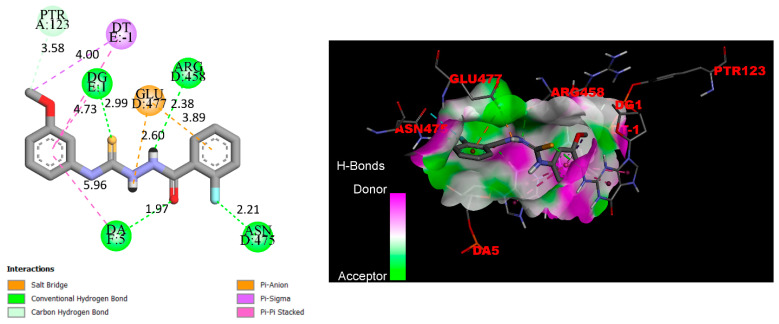
2D and 3D interaction maps of **T4A** with topoisomerase IV (PDB 3LTN).

**T4A** engages in hydrogen bonding within the binding site, interacting with Arg458 (2.38 Å), Asn475 (2.21 Å), and the nucleotides DG1 (2.99 Å) and DA (1.97 Å) (Figure 2). Additionally, it partakes in lipophilic non-covalent interactions, including Pi Sigma, Pi-anion, and Pi-Pi Stacked interactions.

**Figure 3:**
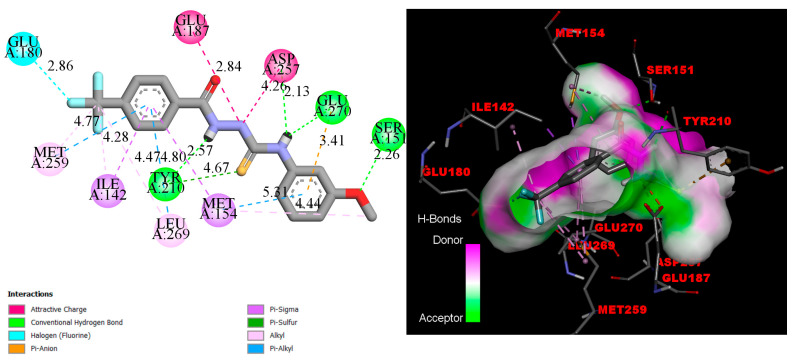
2D and 3D interaction maps of **T9A** with D-alanine—D-alanine ligase (PDB 1IOV).

One of most potent compounds identified through docking studies, **T9A**, exhibits numerous interactions with Ddl, comprising four hydrogen bonds with Tyr210, Asp257, Glu260, and Ser151 (Figure 3). The presence of a 3-methoxy substituent appears to enhance the overall binding energy, contributing to the formation of a hydrogen bond with Ser151. Moreover, the trifluoromethyl group engages in both halogen bonding and lipophilic interactions with Glu180, Met259, Leu269, and Ile142. Additionally, the dual phenyl cores facilitate lipophilic interactions within the channel, involving Met259, Ile142, Leu269, and Met154. Contrary to the findings of Alice Ameryckx et al., who argue for the essential role of a 2-hydroxy substituent in mediating antibacterial activity through Ddl inhibition, our results suggest otherwise [18]. This discrepancy may be attributed to the greater overall distance of our molecule from Ddl, alongside additional binding contributions from the trifluoromethyl substituent. 

## 3. Experiment

### 3.1. Chemistry

#### 3.1.1. General Comments

All of the substances were purchased from Sigma–Aldrich (Munich, Germany) and used without further purification. The ^1^H NMR and ^13^C NMR spectra were recorded on the Bruker Avance 600 (Bruker BioSpin GmbH, Rheinstetten, Germany) in DMSO-d_6_. IR spectra were recorded on Spectrometer FT-IR Nicolett 8700 (Thermo Scientific, Waltham, MA, USA) The melting points were determined on the Stuart SMP50 melting point apparatus (Cole Parmer Ltd., Stone, UK) and are uncorrected. The purity of the compounds and the progress of the reaction were monitored by TLC (aluminum sheet 60 F254 plates (Merck Co., Kenilworth, NJ, USA) using the solvent system CHCl_3_/EtOH (10:2, *v*/*v*). Elemental analyses were determined by a Perkin Elmer 2400 Series II CHNS/O analyzer (Waltham, MA, USA).

Compounds **T2A**, **T3A**, **T6A**, **T7A**, and **T9A** were obtained and described in our previous work [16].

Compound **T1A** was patented previously [17].

#### 3.1.2. Synthesis of Thiosemicarbazide Derivatives

0.01 Mol of the appropriate hydrazide along with 5 mL of 96% ethanol were placed in a round-bottom flask. The mixture was heated using an electric heating cap under a reflux condenser until the hydrazide was completely dissolved and a clear solution was obtained. An equimolar amount of 3-methoxyphenyl isothiocyanate was then added. The mixture was heated at reflux for 1 h. The contents of the flask were then cooled to room temperature. After complete precipitation of the product, the formed precipitate was filtered off and then washed with hot water and diethyl ether. The obtained product was purified by crystallization from 96% ethanol.

Detailed physicochemical properties of new thiosemicarbazides:

1-(2-Fluorobenzoyl)-4-(3-methoxyphenyl)thiosemicarbazide (**T4A**)

CAS 901361-71-5

Yield 64%, m.p. 171–172 °C. Spectral data were as follows: IR (cm^−1^) KBr: 3320 (NH); 1669 (C=O), 1573 (CH_arom_); 1362 (C=S); 1263 (C-O-C). ^1^H NMR (DMSO-d_6_) δ (ppm): 3.75 (s, 3H, CH_3_), 6.75 (d, 1H, CH_arom_, *J* = 8.7 Hz), 7.05 (d, 1H, CH_arom_, *J* = 7.8 Hz), 7.18 (bs, 1H, CH_arom_,), 7.25 (t, 1H, CH_arom_, *J* = 8.1 Hz), 7.33 (d, 1H, CH_arom_, *J* = 7.9 Hz), 7.35–7.36 (m, 1H, CH_arom_), 7.59–7.62 (m, 1H, CH_arom_), 7.85 (bs, 1H, CH_arom_,), 9.84 (bs, 2H, 2NH), 10.33 (s, 1H, NH). ^13^C NMR (DMSO-d_6_) δ (ppm): 55.5; 110.90; 111.8; 116.7 (d, *J* = 22.0 Hz); 118.3; 122.2; 124.4; 129.3; 131.1; 133.7; 140.7; 159.5; 160.1 (d, *J* = 250.5 Hz); 163.9; 181.4. Elemental analysis for C_15_H_14_FN_3_O_2_S. Calculated: C 56.41; H 4.42; N 13.16. Found: C 56.43; H 4.40; N 13.15.

1-(3-Fluorobenzoyl)-4-(3-methoxyphenyl)thiosemicarbazide (**T5A**)

CAS 891103-20-1

Yield 93%, m.p. 172–173 °C. Spectral data were as follows: IR (cm^−1^) KBr: 3319 (NH); 1638 (C=O), 1581 (CH_arom_); 1361 (C=S); 1259 (C-O-C). ^1^H NMR (DMSO-d_6_) δ (ppm): 3.75 (s, 3H, CH_3_), 6.75 (d, 1H, CH_arom_, *J* = 7.2 Hz), 7.04 (d, 1H, CH_arom_, *J* = 8.0 Hz), 7.12 (bs, 1H, CH_arom_,), 7.24 (t, 1H, CH_arom_, *J* = 8.1 Hz), 7.46 (t, 1H, CH_arom_, *J* = 8.5 Hz), 7.59–7.62 (m, 1H, CH_arom_ *J* = 7.7 Hz), 7.77 (d, 1H, CH_arom_ *J* = 9.7 Hz), 7.80 (d, 1H, CH_arom_, *J* = 6.3 Hz), 9.77 (bs, 2H, 2NH), 10.65 (s, 1H, NH). ^13^C NMR (DMSO-d_6_) δ (ppm): 55.5; 111.0; 112.1; 115.2 (d, *J* = 24.0 Hz); 118.5; 119.2 (d, *J* = 20.8 Hz); 124.5; 129.1; 130.9; 135.3; 140.7; 159.4; 162.2 (d, *J* = 243.0 Hz), 165.2; 181.3. Elemental analysis for C_15_H_14_FN_3_O_2_S. Calculated: C 56.41; H 4.42; N 13.16. Found: C 56.42; H 4.39; N 13.13.

4-(3-Methoxyphenyl)-1-(3-trifluoromethylbenzoyl)thiosemicarbazide (**T8A**)

Yield 87% (0.24 g), m.p. 173–175 °C. Spectral data were as follows: IR (cm^−1^) KBr: 3323 (NH); 1670 (C=O), 1573 (CH_arom_); 1365 (C=S); 1264 (C-O-C).^1^H NMR (DMSO-d_6_) δ ppm: 3.75 (s, 3H, CH_3_), 6.76 (d, 1H, CH_arom_, *J* = 8.3 Hz), 7.03 (d, 1H, CH_arom_, *J* = 8.0 Hz), 7.11 (bs, 1H, CH_arom_,), 7.25 (t, 1H, CH_arom_, *J* = 8.1 Hz), 7.78 (t, 1H, CH_arom_, *J* = 7.8 Hz), 7.98 (d, 1H, CH_arom_ *J* = 7.8 Hz), 8.24 (d, 1H, CH_arom_ *J* = 7.8 Hz), 8.30 (s, 1H, CH_arom_), 9.80 (s, 2H, 2NH), 10.82 (s, 1H, NH). ^13^C NMR (DMSO-d_6_) δ (ppm): 55.5; 111.0; 112.2; 118.6; 121.7; 125.1 (d, *J* = 49.4 Hz); 127.1; 128.8; 129.54 (q, *J* = 31.5 Hz); 130.1; 132.4; 134.0; 140.7; 159.4; 165.1, 181.4. Elemental analysis for C_16_H_14_F_3_N_3_O_2_S. Calculated: C 52.03; H 3.82; N 11.38. Found: C 52.00; H 3.79; N 11.36.

### 3.2. Microbiology

The antimicrobial activity of a series of thiosemicarbazides was tested against six Gram-positive reference bacterial strains (*S. aureus* NCTC 4163, *S. aureus* ATCC 25923, *S. aureus* ATCC 6538, *S. aureus* ATCC 29213, *S. epidermidis* ATCC 12228, *S. epidermidis* ATCC 35984) and two Gram-negative reference bacteria (*E. coli* ATCC 25922, *P. aeruginosa* ATCC 15442). The strains of the microorganisms selected for testing are responsible for the most common bacterial infections in humans. To perform the test, all microorganisms were placed in sterile saline (0.85% NaCl) to obtain a suspension with an optical density of 0.5 McFarland standard; 1.50 × 10^8^ colony-forming units (CFU)/mL. For the determination, solutions of the tested compounds with a concentration of 50 mg/mL were prepared using dimethyl sulfoxide (DMSO) as a solvent. Then, the previously prepared solutions were diluted with Mueller–Hinton broth, obtaining samples with a concentration of 2000 µg/mL. In the next step, solutions of the tested compounds were introduced into the wells of the microplate using the two-fold dilution method in broth. In this way, final concentrations from 0.49 to 1000 µg/mL were obtained. In the tests, a negative control without the tested compounds was used. Ciprofloxacin at concentrations of 0.007–15.625 µg/mL was used as a positive control. This antibiotic was chosen because it is one of the most frequently used drugs in outpatient treatment of bacterial infections.

The antimicrobial activity of the tested compounds in vitro was determined using the MIC value, defined as the lowest concentration of the compound at which no visible growth of test organisms was observed. MIC values were determined by the broth microdilution method, following CLSI recommendations [19,20]. MIC determinations were performed in triplicate.

### 3.3. Docking

In our study on antibacterial activity, we employed the same enzymes as those mentioned in the previous article, specifically Topoisomerase IV (PDB ID: 3LTN–Topo IV) [21] and DNA gyrase (PDB ID: 6FQM) [22]. Additionally, we included D-alanine—D-alanine ligase (PDB ID: 1IOV—Ddl) as a target enzyme, due to the identification of thiosemicarbazides as inhibitors in existing scientific literature [23]. The optimization of the three-dimensional shapes of ligands was performed using HyperChem 7.5. Conversion of the generated files was carried out using OpenBabel 3.1.1. The docking protocol employed for the validation and execution of the in silico simulations was consistent with the methodology described in the previous article. The potential inhibitory activity of the compounds against the targeted enzymes was inferred from comparisons of binding energies and inhibition constants (Ki) relative to native ligands from the selected spectra. Molecular interactions and protein–ligand complexes were visualized and analyzed using Discovery Studio Visualizer version 24.1, facilitating a comprehensive assessment of the binding affinities and interactions between the synthesized compounds and their respective enzymatic targets.

## 4. Conclusions

We conducted research that was a continuation of previous work, the aim of which was to search for new compounds with significant antibacterial activity within the group of thiosemicarbazides with a 3-methoxyphenyl substituent. In the course of our research, we obtained a series of compounds differing in the type and position of the substituent on the phenyl ring in position 1 of the thiosemicarbazide system. We tested them for selective strains of Gram-positive and Gram-negative bacteria. All newly synthesized molecules showed no activity against Gram-negative bacteria. Most tested thiosemicarbazide derivatives showed moderate activity against all *S. aureus* strains. The highest antibacterial activity against two bacterial strains of *S. aureus*—*S. aureus* ATCC 25923 and *S. aureus* NCTC 4163—was demonstrated by 1-(2-fluorobenzoyl)-4-(3-methoxyphenyl)thiosemicarbazide **T4A**, with an MIC value of 32 µg/mL against both strains. The compound that showed activity against all tested strains of Gram-positive bacteria, including both *S. aureus* and *S. epidermidis* (with MIC values in the range of 64–128 µg/mL), was **T9A**, a thiosemicarbazide with a 4-trifluoromethylphenyl substituent. Analyzing the results, it can be seen that the trifluoromethylphenyl moiety is an interesting pharmacophore that has a beneficial impact on antibacterial activity within this group of compounds. Based on the obtained molecular docking results, it is not possible to determine the mechanism of action for the presented derivatives, suggesting a multi-directional mechanism of action. The compounds may act by inhibiting gyrase, topoisomerase, and D-alanine—D-alanine ligase.

## Figures and Tables

**Table 1:** Antibacterial data (MIC, μg/mL) for compounds **T1A**–**T9A** and the reference drug.

MIC—Minimal Inhibitory Concentration [µg/mL]											
Microorganism		T1A	T2A	T3A	T4A	T5A	T6A	T7A	T8A	T9A	CIP
***Gram-positive bacteria***	*S. aureus* NCTC 4163	>256	>256	64	32	128	256	64	64	64	0.125
	*S. aureus* ATCC 25923	>256	>256	64	32	128	256	64	64	64	0.25
	*S. aureus* ATCC 6538	>256	>256	64	64	128	256	64	128	64	0.125
	*S. aureus* ATCC 29213	>256	>256	128	64	128	256	128	128	64	0.5
	*S. epidermidis* ATCC 12228	>256	>256	>256	128	256	256	>256	256	64	0.25
	*S. epidermidis* ATCC 35984	>256	>256	>256	256	256	256	>256	256	128	0.125
***Gram-negative*** ***bacteria***	*E. coli* ATCC 25922	>256	>256	>256	>256	>256	>256	>256	>256	>256	0.008
	*P. aeruginosa* ATCC 15442	>256	>256	>256	>256	>256	>256	>256	>256	>256	0.125

CIP—ciprofloxacin.

**Table 2:** Antibacterial data (MIC, μg/mL) for compounds **T1A**–**T9A** and **T1B**–**T9B**.

** 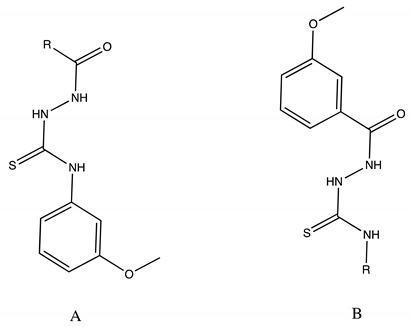 **		
**Compounds**	***S. aureus*** ***ATCC 25923***	***S. epidermidis*** ***ATCC 12228***
	**MIC [µg/mL]**	**MIC [µg/mL]**
**T1A**	**>256**	**>256**
T1B	62.5	62.5
**T2A**	**>256**	**>256**
T2B	1000	500
**T3A**	**64**	**>256**
T3B	1000	125
**T4A**	**32**	**256**
T4B	1000	1000
**T5A**	**128**	**256**
T5B	125	>1000
**T6A**	**256**	**256**
T6B	500	250
**T7A**	**64**	**>256**
T7B	250	62.5
**T8A**	**64**	**256**
T8B	250	31.25
**T9A**	**64**	**128**
T9B	500	125

**Table 3:** AutoDock docking scores of the tested compounds and the reference ligands.

Compounds	Topoisomerase IV (3LTN)		DNA Gyrase (6FQM)		Ddl (1IOV)	
	Binding Energy Kcal/mol	Inhibition Constant, Ki uM	Binding Energy Kcal/mol	Inhibition Constant, Ki uM	Binding Energy Kcal/mol	Inhibition Constant, Ki uM
**T1A**	−8.32	0.79	−7.84	1.79	−8.73	0.39
**T2A**	−7.86	1.85	−8.73	0.39	−7.83	1.83
**T3A**	−8.03	1.3	−9.92	0.053	−8.64	0.46
**T4A**	−8.55	0.15	−9.78	0.068	−8.99	0.26
**T5A**	−6.59	14.87	−6.64	13.50	−8.88	0.31
**T6A**	−7.36	4.03	−8.48	0.20	−8.19	0.99
**T7A**	−6.64	13.50	−8.67	0.45	−9.03	0.24
**T8A**	−7.83	1.82	−9.85	0.60	−8.78	0.35
**T9A**	−8.48	0.19	−9.50	0.11	−9.55	0.10
PD 0305970	−8.38	0.72	-	-	-	-
E32	-	-	−9.29	0.155	-	-
POV	-	-	-	-	−9.50	0.11

## Data Availability

Data are contained within the article and Supplementary Materials.

## Supplementary Materials

The following supporting information can be downloaded at: https://www.mdpi.com/article/10.3390/molecules29061333/s1.
