# Supplementary material for: The Importance of Substituent Position for Antibacterial Activity in the Group of Thiosemicarbazide Derivatives
Source: Molecules. 2024 Mar 17;29(6):1333. doi: 10.3390/molecules29061333 (PMC10974262; doi:10.3390/molecules29061333)
Supplement: Supplementary file 1 [file molecules-29-01333-s001.zip › molecules-2916790-supplementary.pdf]

Supplementary Information

The Importance of Substituent Position for Antibacterial Activity in the Group of Thiosemicarbazide Derivatives

Sara Janowska [1,*], Joanna Stefańska [2], Dmytro Khylyuk [3] and Monika Wujec [3,*]

[1] Department of Pathobiochemistry and Interdisciplinary Applications of Ion Chromatography, Biomedical Sciences, Medical University of Lublin, 1 Chodzki Street, 20-093 Lublin, Poland

[2] Department of Pharmaceutical Microbiology, Centre for Preclinical Research, Medical University of Warsaw, Banacha 1B Street, 02-097 Warsaw, Poland; joanna.stefanska@wum.edu.pl

[3] Department of Organic Chemistry, Faculty of Pharmacy, Medical University, 4a Chodzki Street, 20-093 Lublin, Poland; dmytro.khylyuk@umlub.pl

* Correspondence: sara.janowska@umlub.pl (S.J.); monika.wujec@umlub.pl (M.W.)

Table of contents:

^{1}H NMR spectra of thiosemicarbazide derivatives ……………………………………………………………………..………………………….…………2
^{13}C NMR spectra of thiosemicarbazide derivatives……..…………………………………………………………………….…………………….…….….5
IR spectra of thiosemicarbazide derivatives……………………………………….……..…………………………….………………………………….7

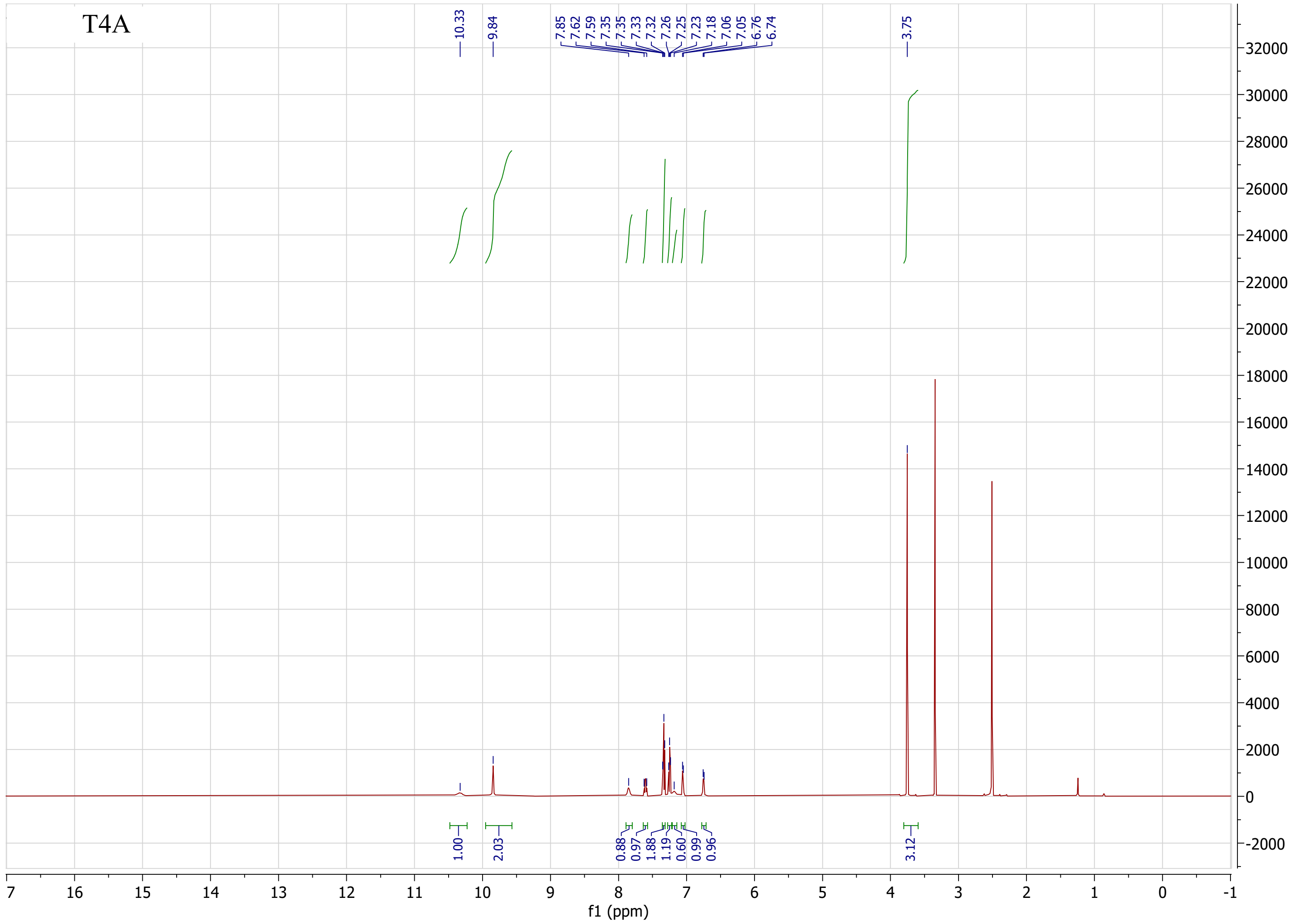

Figure S1. The ^{1}H NMR of compound T4A.

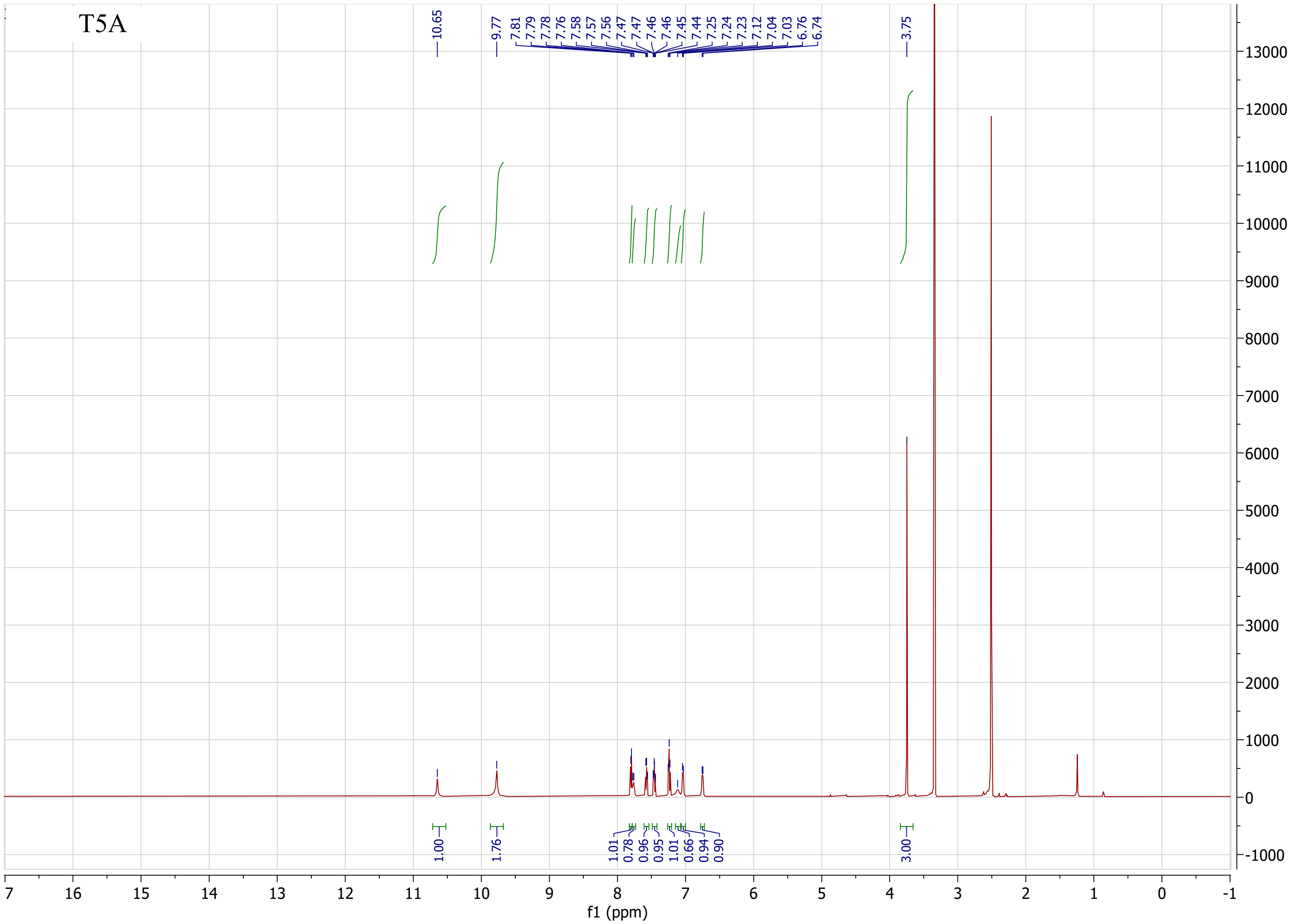

Figure S2. The ^{1}H NMR of compound T5A.

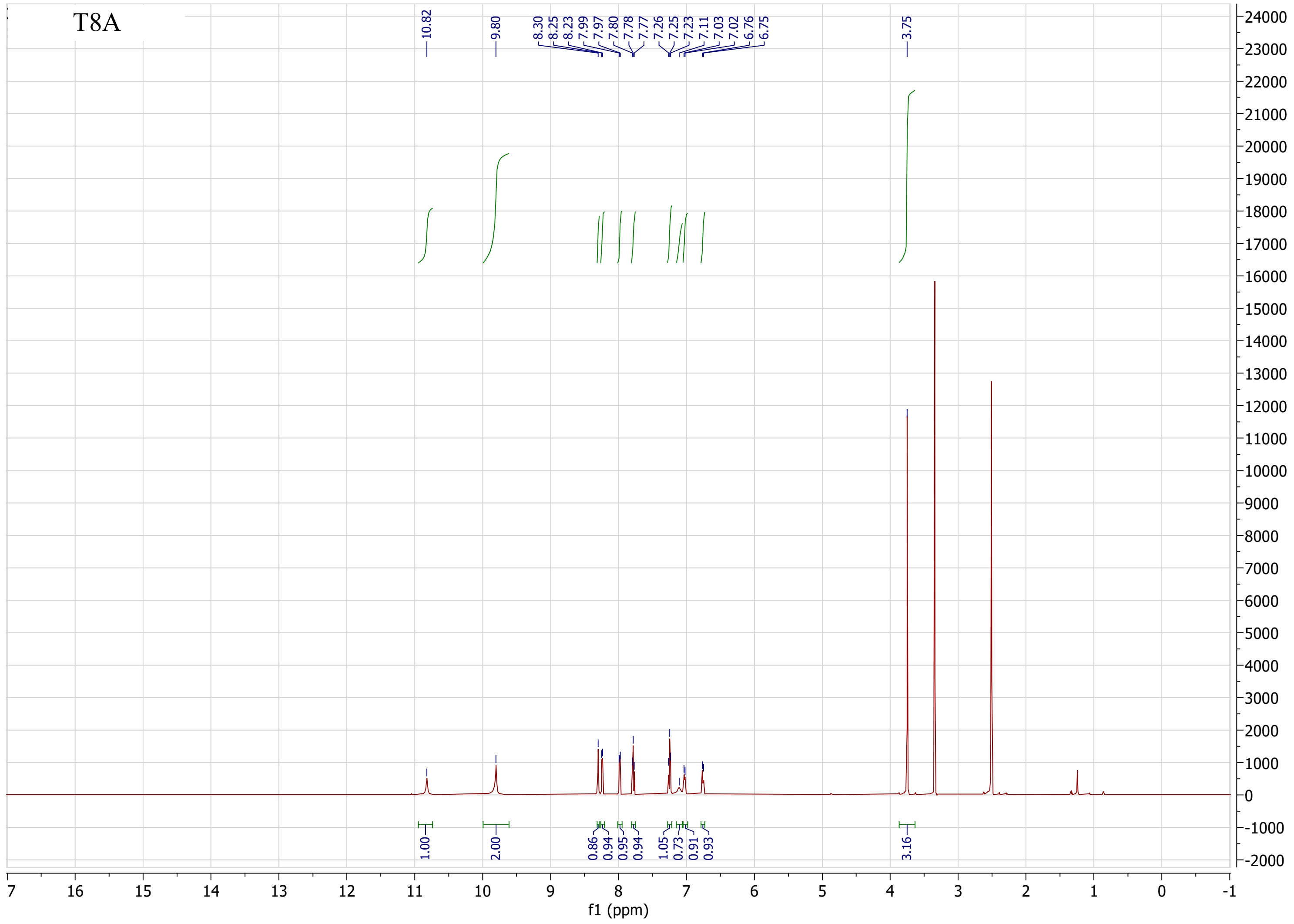

Figure S3. The ^{1}H NMR of compound T8A.

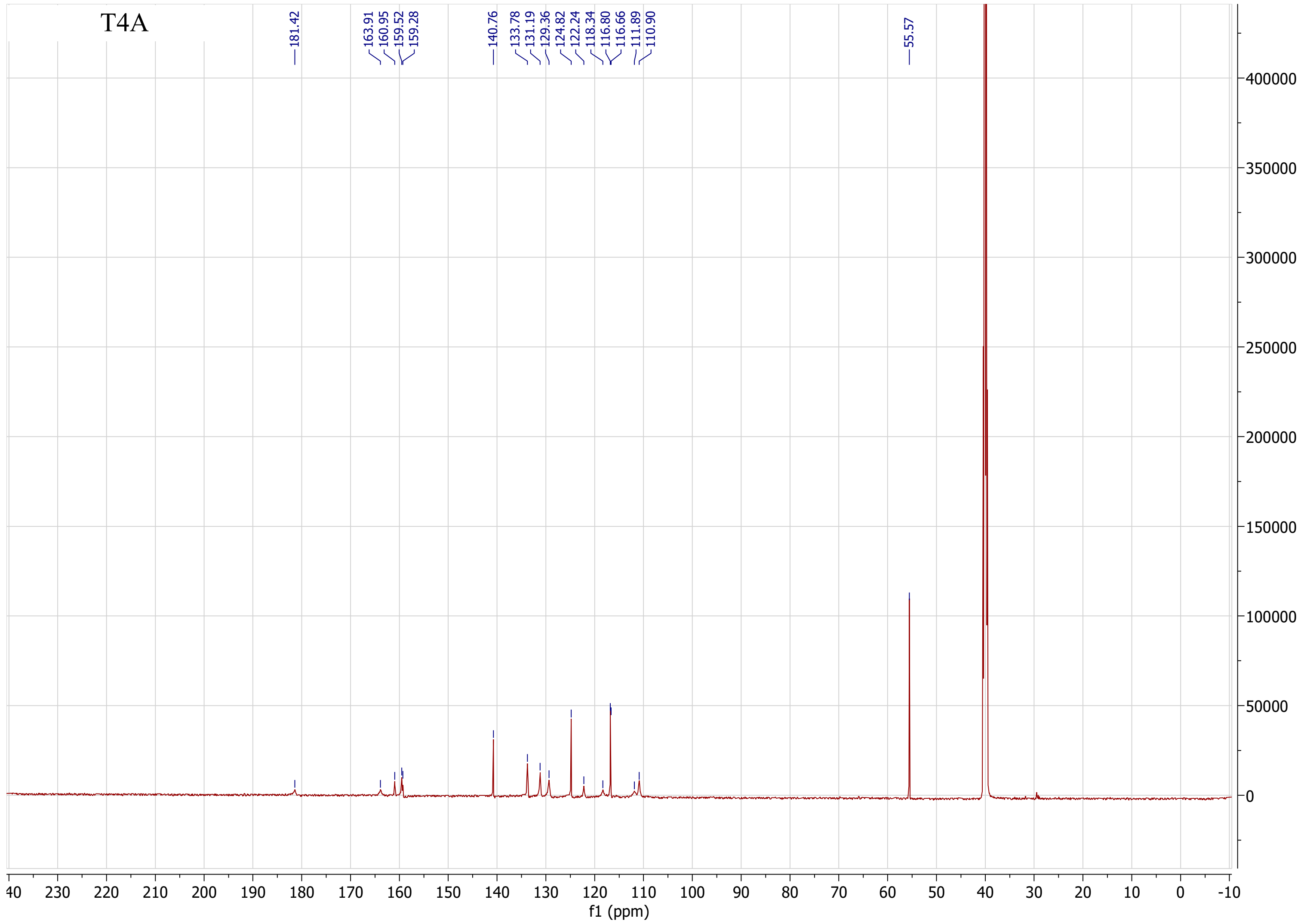

Figure S4. The ^{13}C NMR of compound T4A.

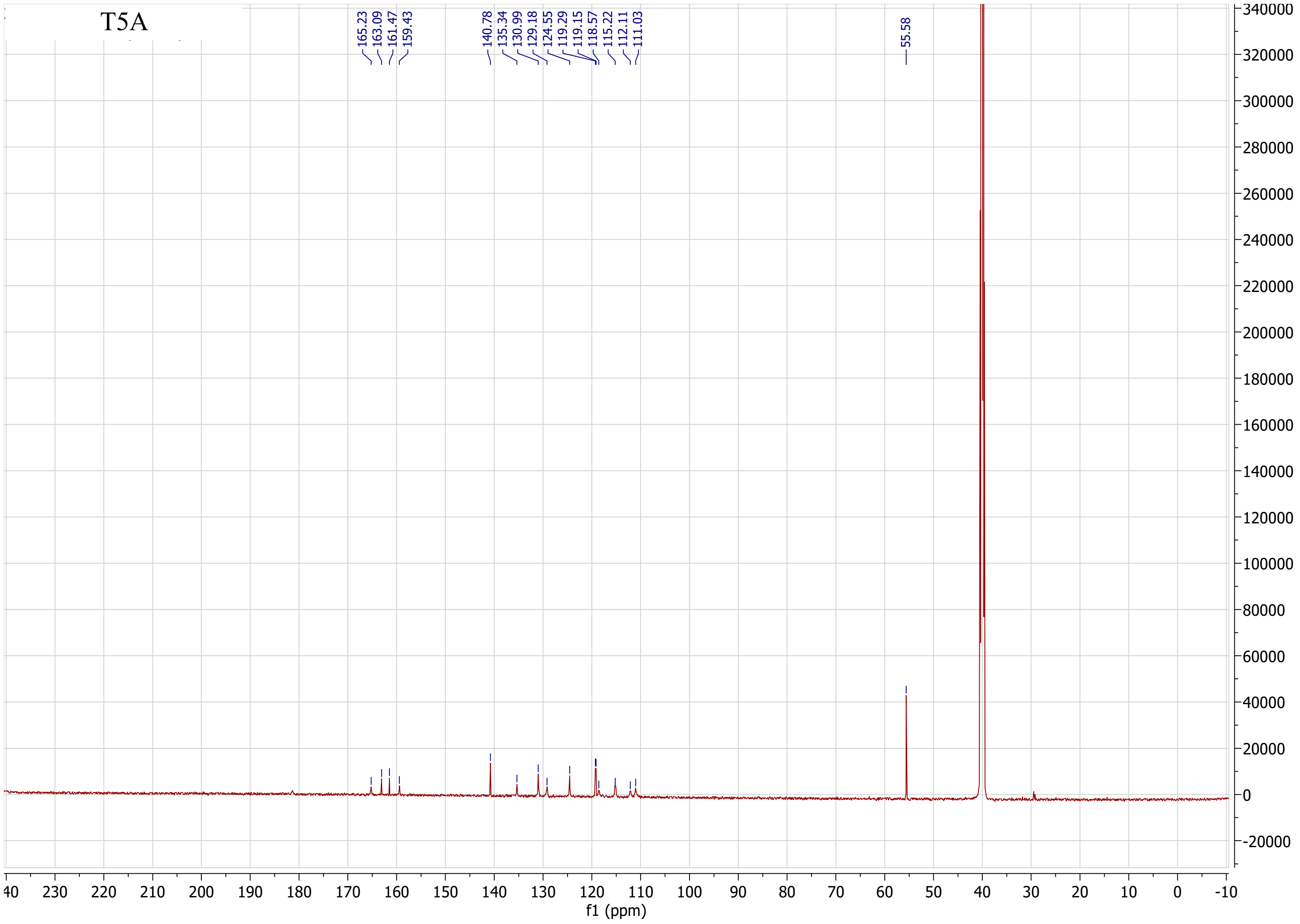

Figure S5. The ^{13}C NMR of compound T5A.

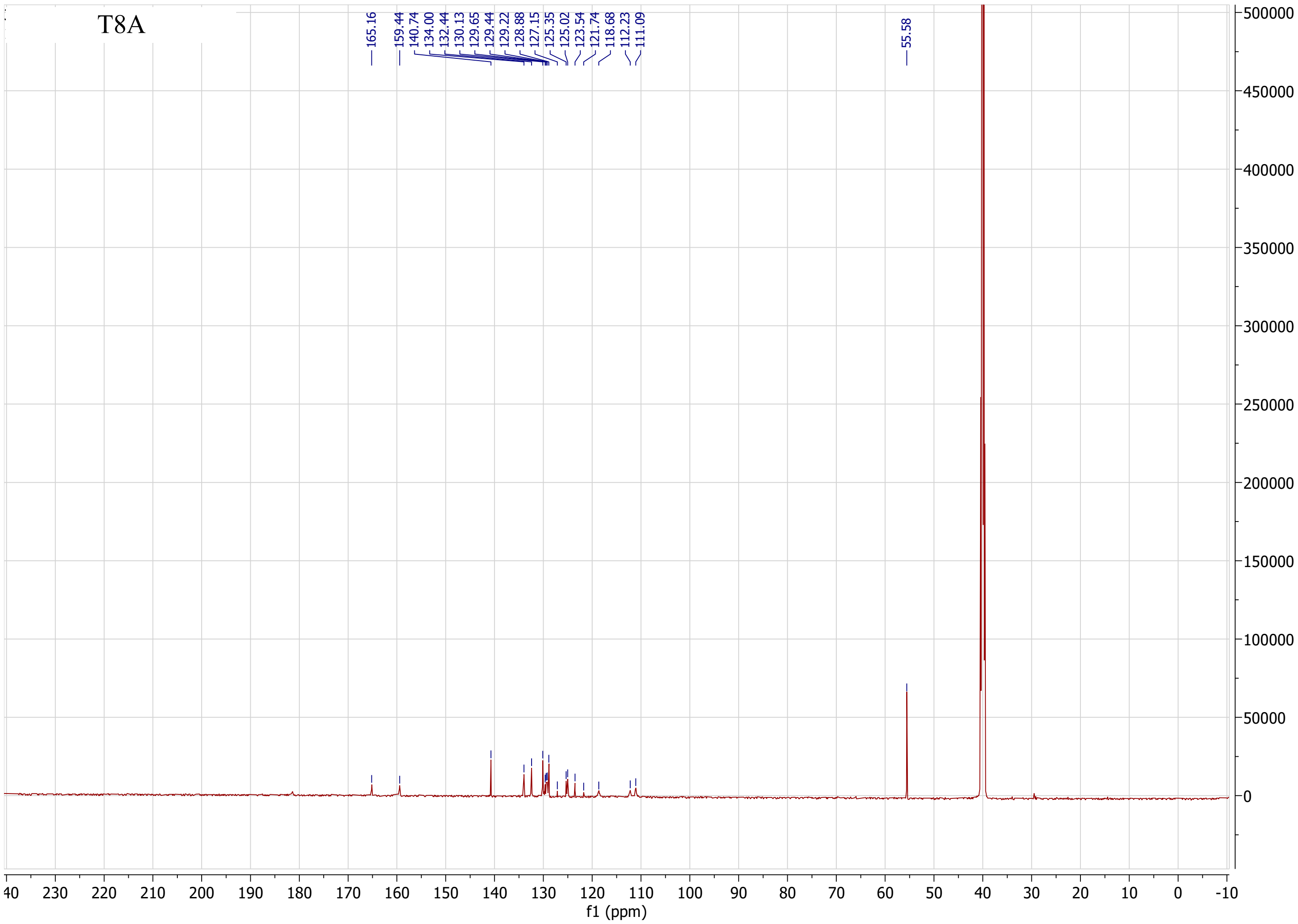

Figure S6. The ^{13}C NMR of compound T8A.

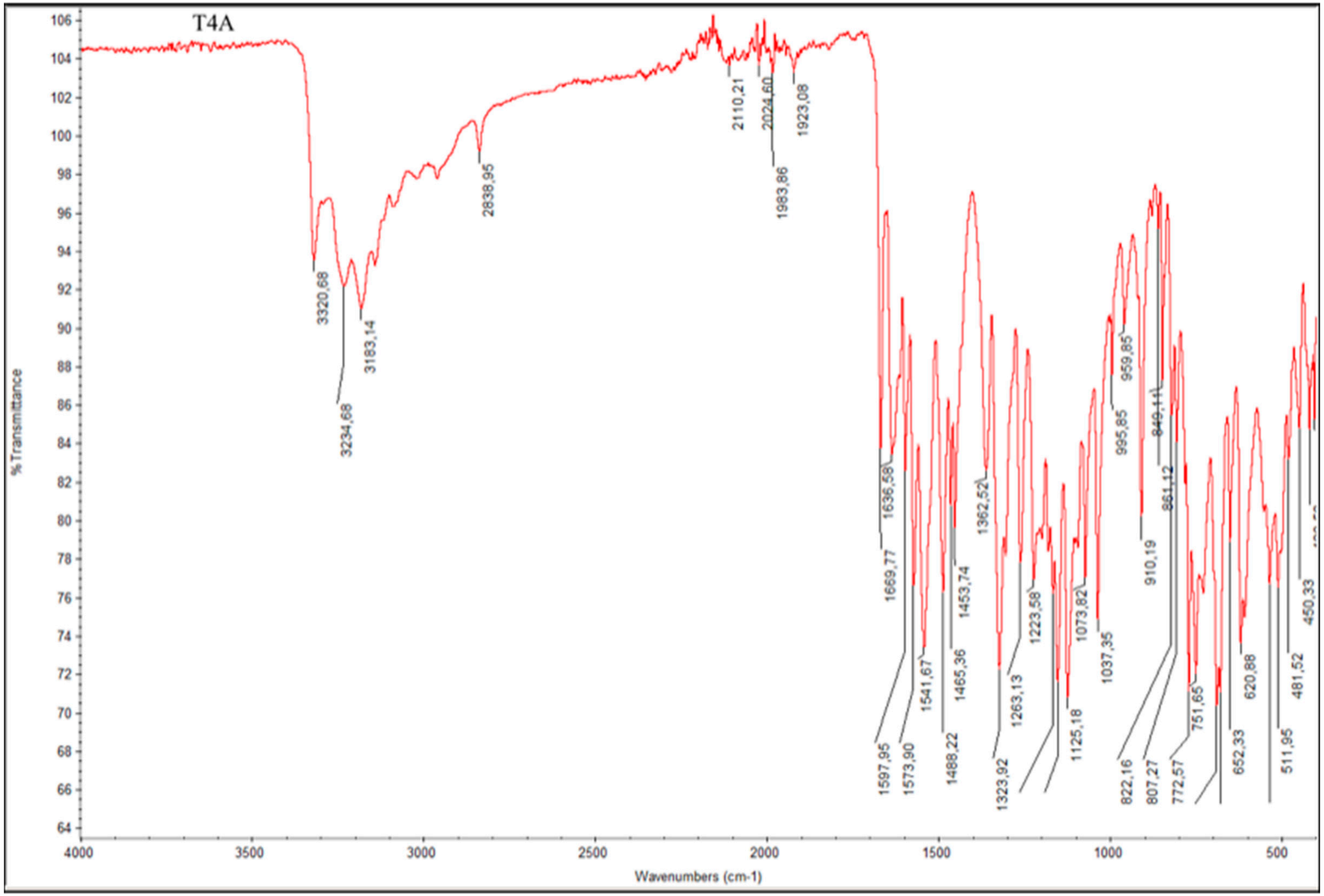

Figure S7. The IR of compound T4A.

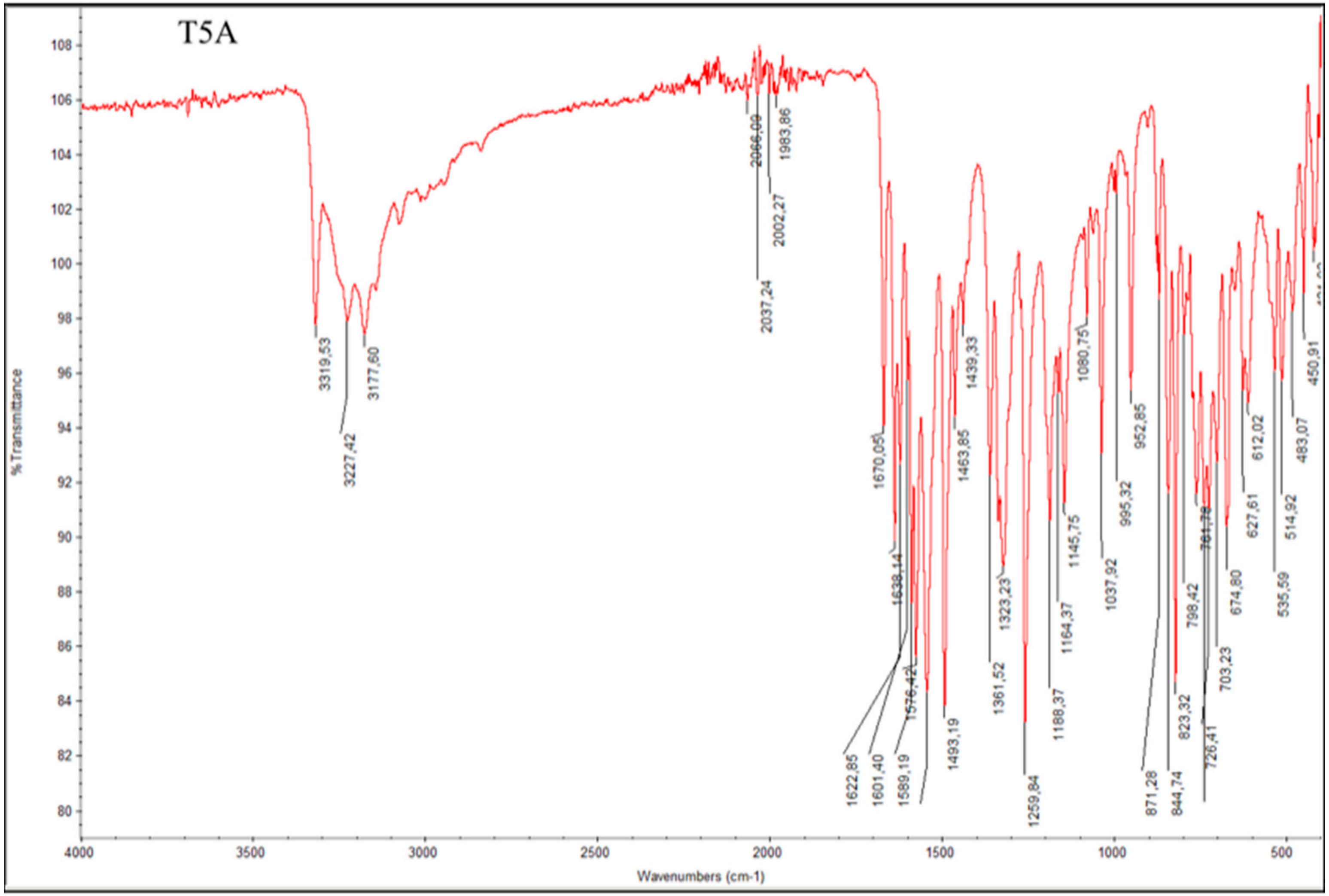

Figure S8. The IR of compound T5A.

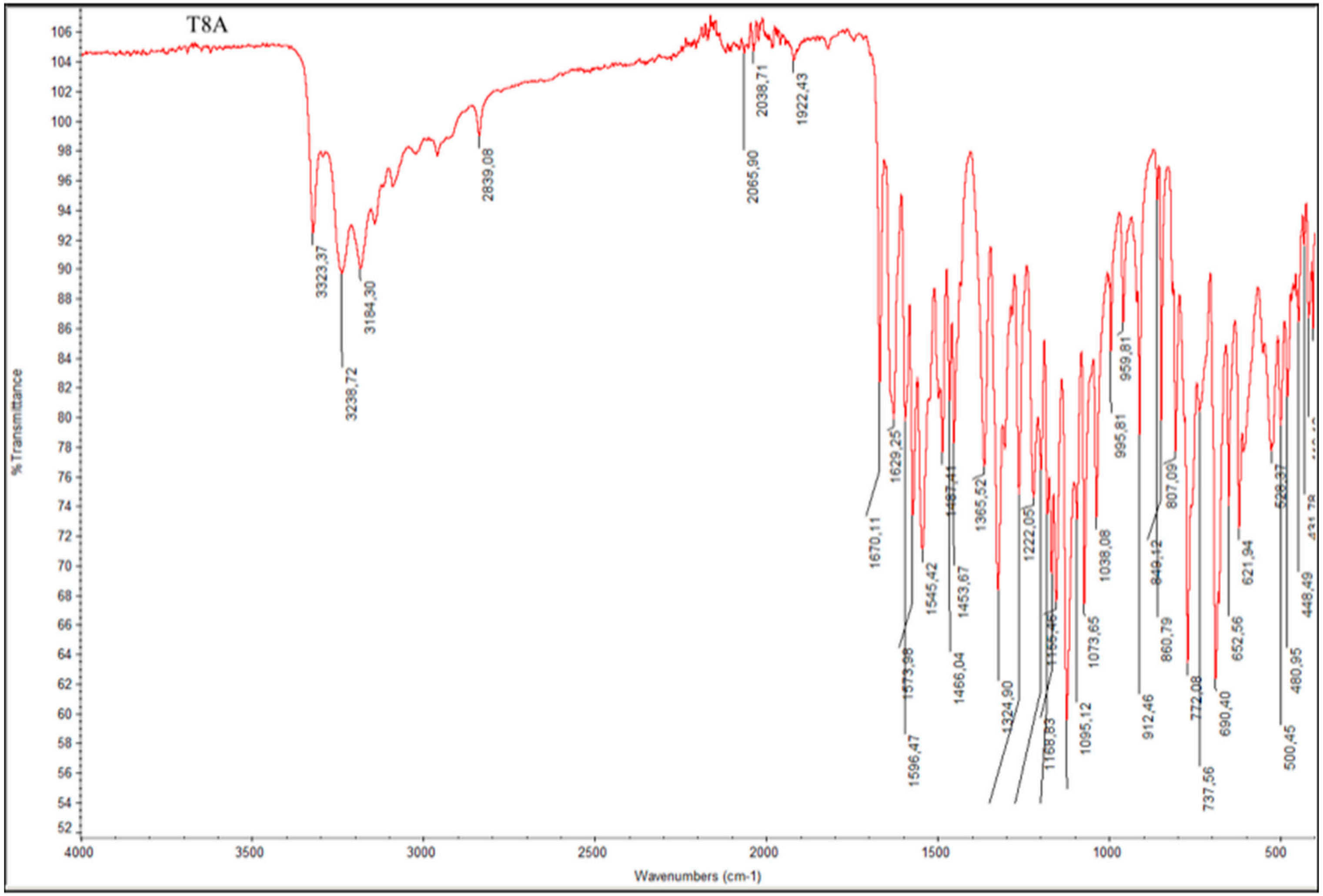

Figure S9. The IR of compound T8A.
